# Putting RFMix and ADMIXTURE to the test in a complex admixed population

**DOI:** 10.1186/s12863-020-00845-3

**Published:** 2020-04-07

**Authors:** Caitlin Uren, Eileen G. Hoal, Marlo Möller

**Affiliations:** DSI-NRF Centre of Excellence for Biomedical Tuberculosis Research, South African Medical Research Council Centre for Tuberculosis Research, Division of Molecular Biology and Human Genetics, Faculty of Medicine and Health Sciences, Stellenbosch University, Room 4036, 4th Floor Education Building, Francie van Zijl Drive, Cape Town, 8000 South Africa

**Keywords:** South Africa, Local ancestry inference, Population genetics, RFMix, ADMIXTURE

## Abstract

**Background:**

Global and local ancestry inference in admixed human populations can be performed using computational tools implementing distinct algorithms. The development and resulting accuracy of these tools has been tested largely on populations with relatively straightforward admixture histories but little is known about how well they perform in more complex admixture scenarios.

**Results:**

Using simulations, we show that RFMix outperforms ADMIXTURE in determining global ancestry proportions even in a complex 5-way admixed population, in addition to assigning local ancestry with an accuracy of 89%. The ability of RFMix to determine global and local ancestry to a high degree of accuracy, particularly in admixed populations provides the opportunity for more accurate association analyses.

**Conclusion:**

This study highlights the utility of the extension of computational tools to become more compatible to genetically structured populations, as well as the need to expand the sampling of diverse world-wide populations. This is particularly noteworthy as modern-day societies are becoming increasingly genetically complex and some genetic tools and commonly used ancestral populations are less appropriate. Based on these caveats and the results presented here, we suggest that RFMix be used for both global and local ancestry estimation in world-wide complex admixture scenarios particularly when including these estimates in association studies.

## Background

Admixture, the exchange of genetic material between distinct populations, is a hallmark of modern society - it can occur between closely or distantly related populations [1]. This exchange of genetic material leads to population structure; the pattern, timing and extent has been investigated in detail in a number of populations [1–3]. Such studies on southern African populations are particularly noteworthy as this area is postulated to be the geographical origin of modern humans [4]. Furthermore, it was the final destination of various human population migrations that have resulted in a unique pattern of genetic diversity in the region [3, 5–8]. Therefore, investigating population structure in modern southern African populations may reveal more about the area’s rich history.

Correctly and efficiently determining ancestral proportions in an admixed population is possible by using computational and statistical algorithms that adapt to a variety of demographic scenarios [9–11]. The ability to determine the ancestral origin of a particular chromosomal region (or the overall admixture proportions) in an admixed individual has enabled the mapping of the origins of genetic risk factors in complex disease [12–14]. The majority of the computational and statistical tools used for global and local ancestry inference (GAI and LAI respectively) were however tested on and tailored to 2- and 3-way admixed populations (such as African Americans, Hispanics and Latino’s). The extension to more complex admixed populations and the evaluation of the resulting accuracy has yet to be investigated.

As with most geographical regions, southern Africa houses a multitude of diverse human populations that all share in the migratory history in the area. One of the most unique populations in southern Africa is the South African Coloured (SAC) population (as termed in the South African census). The SAC population received ancestral contributions from 5 distinct populations; Bantu-speaking African (~ 30%), KhoeSan (~ 30%), European (~ 20%), East Asian (~ 10%) and South East Asian populations (~ 10%) [3, 5–8]. The admixture began approximately 15 generations ago and followed a continuous migration model [3]. The extent, mode and timing of admixture events is unique and creates a highly complex population with heterogenous ancestral haplotypes and linkage disequilibrium patterns.

The first step in a study design aimed at characterizing a relationship between ancestry and disease (such as genome-wide association studies and admixture mapping) is to understand the ancestral composition of the study population. Here we have set out to test the accuracy of global and local ancestry inference in one of the most complex admixed populations world-wide, using newly available dense genotyping data. A simulated 5-way admixed population is generated, and global and local ancestry estimates are compared to the true values to determine the accuracy of the computational algorithm.

## Results

The aim of this study was to determine the accuracy of global and local ancestry inference (GAI and LAI respectively) in one of the most complex populations world-wide- putting it to the ultimate test. In order to do this, a highly complex 5-way admixed population was simulated. The GAI and LAI estimates were then compared to the true simulated data.

### GAI accuracy

A 5-way admixed southern African population was simulated. The average ancestry proportions across these individuals were in line with what is seen in the real-world (Table 1) [3]. The simulations provided the basis with which the global ancestry proportions as calculated by ADMIXTURE [11] and RFMix [9] could be compared.

**Table 1:** Average admixture proportions

	Previously Reported (Uren et al. 2016) (%)	Simulation (%)	ADMIXTURE (unsupervised) (%)	ADMIXTURE (supervised) (%)	RFMix (%)
Bantu-speaking African	32	26 (95% CI: 25–28)	33 (95% CI: 32–35)	31 (95% CI: 30–33)	27 (95% CI: 26–30)
KhoeSan	30	33 (95% CI: 31–36)	25 (95% CI: 23–27)	34 (95% CI: 31–37)	33 (95% CI: 30–36)
European	19	23 (95% CI: 21–25)	26 (95% CI: 24–29)	21 (95% CI: 19–24)	22 (95% CI: 20–24)
East Asian	7	6 (95% CI: 5–9)	7 (95% CI: 5–9)	6 (95% CI: 5–8)	6 (95% CI: 5–9)
South East Asian	12	12 (95% CI: 10–15)	9 (95% CI: 8–12)	8 (95% CI: 7–11)	12 (95% CI: 10–14)

**Fig. 1:**
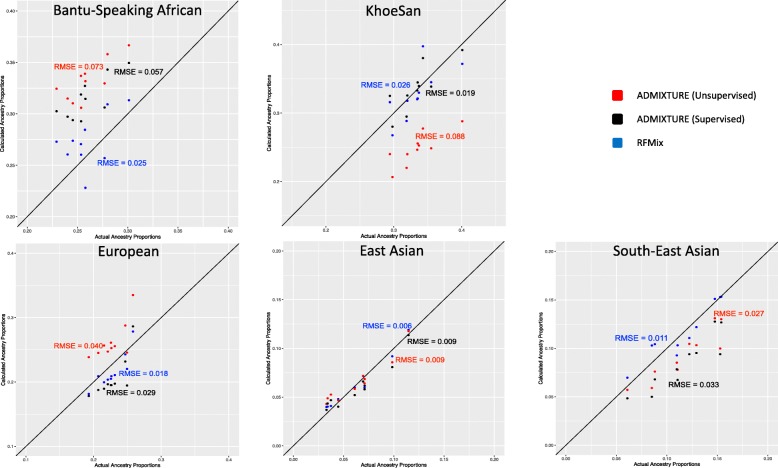
Comparison between observed global ancestry proportions and “true” proportions showing RFMix performs more accurately than ADMIXTURE in ancestry determination. *Admixture proportions calculated by ADMIXTURE are in red (Unsupervised) and black (Supervised), and RFMix in blue. Root Mean Square Errors for every comparison are shown*

Supervised and unsupervised admixture analysis of the simulated dataset by ADMIXTURE and that performed by RFMix, confirmed that the simulated 5-way admixed population is highly heterogenous. Average ancestral proportions for both computational tools are given in Table 1. The comparisons across the 5 ancestries for each simulated individual are also depicted in Fig. 1. Root Mean Squared Errors (RMSE) were calculated for each comparison. As per the RMSE’s, RFMix outperforms both ADMIXTURE runs (unsupervised and supervised) in correctly estimating admixture proportions in the 5-way admixed population, with the exception of KhoeSan ancestry where the accuracy is largely equal. Both ADMIXTURE runs over-estimates the Bantu-speaking African contribution and under-estimates the KhoeSan ancestral proportions. Similarly, the unsupervised ADMIXTURE run overestimates European ancestry and underestimates South East Asian ancestry.

### LAI accuracy

**Fig. 2:**
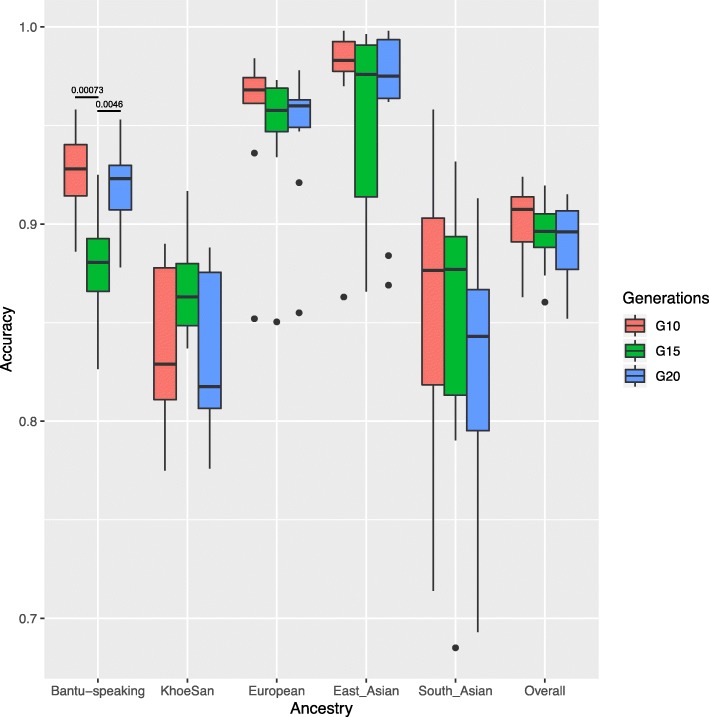
Boxplot showing the robustness of RFMix when using inaccurate time since admixture estimates. *Time since admixture of 10 (red), 15 (green) and 20 (blue) generations are shown. The median (bold horizontal line) and the upper and lower quartiles are shown. Data outside this range are plotted as outliers. The differences in accuracies across generations for each ancestry were assessed using a Wilcoxon non-parametric test. All statistically significant p values (< 0.01) are shown*

Beyond global ancestry proportions, the simulation of a 5-way admixed population resulted in known local ancestry tracts, to which calls by a computational tool can be compared. The ancestral origin of each parental chromosomal region was determined using RFMix. RFMix has been shown to outperform other computational tools in the estimation of local ancestry in complex admixture scenarios [13]. The local ancestry calls by RFMix were compared to the “true” simulated ancestral origin of each region. To determine the robustness of RFMix when inaccurate admixture timing estimates are available, we selected 10, 15 and 20 generations as input for time since admixture. Although there were differences in the accuracy of RFMix when the time since admixture was varied, these differences were not significant (except for Bantu-speaking ancestry) and the direction of these differences varied for each ancestral population (Fig. 2). For this reason, a time since admixture in line with the simulated population was used for further analyses (15 generations).

**Fig. 3:**
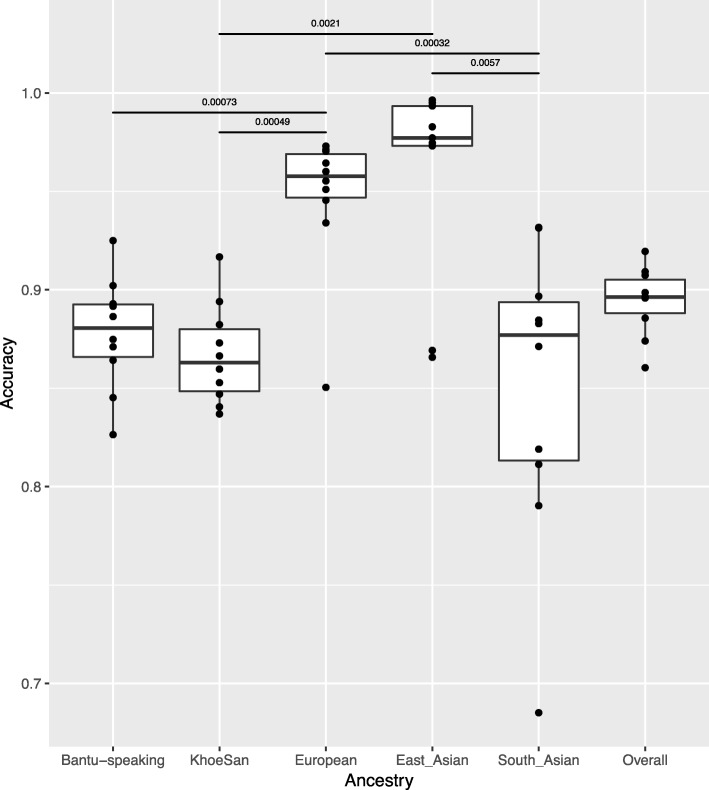
Boxplot showing the accuracy with which RFMix assigns an ancestral origin to a genetic region, stratified by reference population. *The median (bold horizontal line) and the upper and lower quartiles are shown. Data outside this range are plotted as outliers. The differences in accuracies across ancestries were assessed using a Wilcoxon non-parametric test. All statistically significant p values (< 0.01) are shown*

The overall LAI accuracy across all ancestries (15 generations) is ~ 89%; 88% accurate in calling Bantu-speaking African ancestry, 87% calling KhoeSan ancestry, 95% calling European ancestry, 86% calling East Asian ancestry and 85% calling South East Asian ancestry. The statistical significance of RFMix’s ability to call a specific ancestry over another was assessed (Fig. 3). RFMix is able to call East Asian and European ancestry more precisely than any of the other ancestries (Fig. 3).

## Discussion

From the results, we note that the estimates obtained for ADMIXTURE is greatly influenced by the inclusion of admixed reference populations. For example, the undercalling of South East Asian ancestry is most likely due to inherent European ancestry present in South East Asian populations and likewise, Bantu-speaking ancestry in the KhoeSan population. This is consistent with the trends seen in the LAI accuracy analysis. This highlights the need for further improvement in computational tools to distinguish between intra-continental ancestral populations, particularly in Africa as well as to perhaps tailor these tools to complex admixture scenerios where admixture with particular ancestral populations occurred at different times [9]. This is particularly noteworthy as most modern-day populations are admixed and therefore computational tools should be able to account for this within the algorithms.

The evident difference in accuracy estimating admixture proportions using RFMix and ADMIXTURE can be attributed to a number of aspects. RFMix is able to harness a multitude of prior information in order to perform LAI (and therefore GAI) such as LD, relatedness and phase information. Overall, we hypothesize that the addition of this information allows for the increased accuracy of RFMix over ADMIXTURE. Additionally, it is interesting to note that ADMIXTURE’s unsupervised algorithm which is used to tease out fine-scale population structure in admixed populations, performed poorly in relation to the supervised algorithm. This is significant since it is the most widely used ADMIXTURE algorithm and highlights the necessity to move away from estimations not based on haplotypes.

We tested the robusteness of RFMix in calling local ancestry tracts when the incorrect time since admixture was used in the model. In our simulated dataset, we did not find any statistical differences when using an inflated or deflated time since admixture, with the excpetion of calling Bantu-speaking ancestry. This apparent decrease in accuracy using a time since admixture of 15 generations is largely a by-product of the increased accuracy seen when calling KhoeSan ancestry; similar to the trend seen in the GAI accuracy results. Studies that under- or overestimate the time since admixture as well as include admixed ancestral populations that are genetically similar, may experience similar trends with LAI accuracy values across ancestral populations i.e. calling tracts of one ancestral population accurately might decrease the accuracy of another ancestral population that is closely related. One way to pre-empt this would be to incorporate (within the computational algorithm) the specification of a time since admixture for each ancestral population.

## Conclusion

In conclusion, the findings presented here is the first of its kind to detail the accuracy of LAI and GAI in one of the most complex populations worldwide. Due to the accuracy and versatility of RFMix which harnesses prior information as LD, relatedness and phase information in determining global and local ancestry in a single program, it should be the algorithm of choice to characterize more complex admixture scenarios. The inclusion of accurate admixture proportions as a covariate in association studies is vital, and it is our opinion that researchers studying complex admixed populations should use RFMix for this purpose.

Furthermore, we demonstrate that computational tools *are* able to decipher the complex African genetic history with a high degree of accuracy, but there is still some room for improvement regarding the tailoring of computational tools to handle intra-continental, admixed reference and target populations.

As populations become increasingly mobile, the likelihood of admixture between diverse groups is greater. Therefore the extension of these and future computational tools to genetically complex populations from across the world is vital and. The conclusions of this study are therefore relevant and generalizable to other admixed populations.

## Methods

### Data merging and filtering

KhoeSan genotype data from Martin and colleagues [15] was merged with the genetic data generated as part of the Population Architecture using Genomic and Epidemiology dataset [16] and genetic data from the Gujarati Indian and British populations from the 1000 Genomes Project [17]. Preliminary data filtering included a filter for minor allele frequency (0.003), missingness per genotype (max 0.05) and missingness per individual (max 0.01). A total of ~ 776 k SNPs passed these filters and formed the initial merged dataset. Further data filtering is described in the appropriate sections below. Data was phased using SHAPEIT2 utilizing the published African American HapMap recombination map [18, 19]. Populations in the final dataset are summarised in Table 2.

**Table 2:** Population characteristics of the final merged dataset

Population	Number of individuals included
KhoeSan (Nama and ≠Khomani San)	284
European (British)	79
African (Yoruba and Luhya)	35
East Asian (Han)	50
South East Asian (Gujarati)	103

### Simulations

**Fig. 4:**
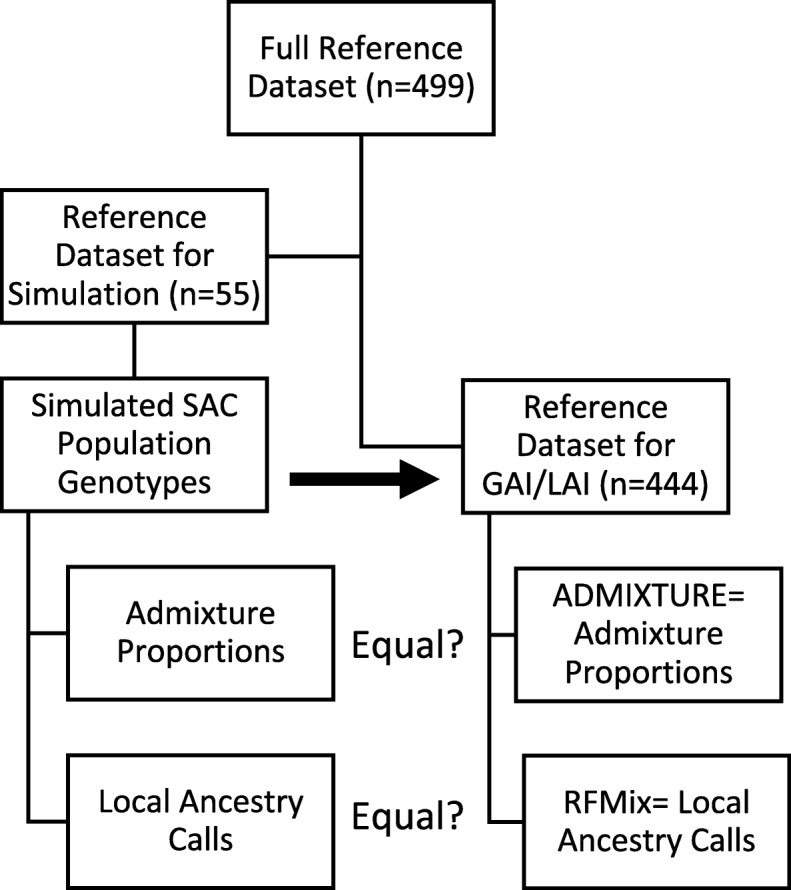
Computational workflow. *The full dataset (n = 499) was divided into a dataset used for the simulation (n = 55) and a dataset used for GAI and LAI (n = 444). Once the simulated SAC population was generated (including global and local ancestry estimations), these true values were compared to values emanating from ADMIXTURE and RFMix. For details, please see the methods section*

The computational workflow is summarised in Fig. 4. A random subset of 55 reference individuals from the final merged dataset described in Table 2 was used to generate a simulated dataset using admix-simu (11 per reference population) [20]. The remaining 444 reference individuals formed the reference dataset for GAI and LAI. A demographic model consisting of specific ancestry proportions and timing of migration, leading a continuous migration model initializing at 15 generations ago, was used to generate a simulated 5-way admixed population [3] (please see Table S1 for the specific admixing proportions). This simulation results in a heterogenous population, reminiscent of a real-world SAC population (see Table 1).

The simulation does not take post-admixture selection into account since it is highly unlikely that 350 years would result in distinct selection signals, rather, the inherent selection signals in the source populations will be transferred in a random manner to the simulated admixed population (adaptive introgression). Genotype as well as local ancestry calls were generated for this simulated dataset from real reference haplotypes, thus capturing the complexity of this heterogenous 5-way admixed South African population.

### Software choices

Although there are a number of software programs that are able to estimate global ancestry (BAPS [21], HAPMIX [22], LAMP [23], FRAPPE [24], sNMF [25] etc), ADMIXTURE is however the most utilized. Reasons for this include the ability to include related individuals in one run and to generate accurate admixture proportions using relatively low-density SNP-array data [11]. The other widely used global ancestry algorithm, STRUCTURE has been shown to overestimate admixture proportions in even simple admixture scenarios, therefore given the demographic history of the population presented here, this software was not used [26].

RFMix was chosen as the local ancestry inference algorithm of choice as it allows for parameter optimization given the number of ancestral populations and the ability to perform LAI in populations more than 2-way or 3-way admixed (limitations of LAMP [23] and HAPMIX [22]). In addition, RFMix has the inherent ability to calculate local and global ancestry simultaneously and allows for array-based input data as well as whole genome sequencing data. Furthermore, initial results by Daya and colleagues suggested that RFMix is the most accurate tool for local ancestry estimation (over and above that calculated for LAMP-LD [27, 28]) in admixed southern African populations however, only a 3-way admixture scenario was tested (San, Bantu-speaking and non-African) [13].

### GAI accuracy

Reference individuals not included in the dataset used for the simulation, were allocated to the dataset used for GAI and LAI. Global ancestry proportions were determined by ADMIXTURE [11] and RFMix [9]

The ADMIXTURE analysis was performed in a supervised and unsupervised manner after filtering the dataset for linkage disequilibrium as per the manual’s recommendations (50 kb window size, step size of 10 kb and R^2^ threshold of 0.1). The supervised algorithm allows for the input of know ancestral origins of the reference individuals whereas the unsupervised algorithm infers the ancestry of all individuals given as input.

RFMix was run using default parameters, a time since admixture of 15 generations (in line with the simulation) as well as 3 expectation-maximization (EM) iterations (further EM iterations were not shown to increase accuracy [9]). The correlation of the two methods by means of the Root Mean Squared Error (RMSE) was performed in R.

### LAI accuracy

Local ancestry calls were generated by RFMix using the same parameters as described in the previous section. The ability to correctly assign local ancestry was calculated in two ways, at an individual level. The first determined the global accuracy using the formula $\frac{d_c\ }{d_t}$, where *d*_*c*_ is the number of sites that had a called ancestry and *d*_*t*_ is the number of sites that had a correctly called ancestry as compared to the simulations. The second method of accuracy estimation looked at this accuracy per ancestral population using the formula $\frac{d_{ca}\ }{d_a}$ where *d*_*a*_ is the number of sites that had a called ancestry and *d*_*ca*_ is the number of sites that the specific ancestry was correctly called [29]. These accuracy estimators were then averaged over all individuals in the simulated 5-way admixed dataset.

## Supplementary information

**Additional file 1: Table S1.** Demographic model used to simulated SAC population

## Data Availability

No new genetic data was generated for this study however all reference data supporting the findings of this study are available via the original publication. The 1000 Genomes data can be access accessed by visiting https://www.internationalgenome.org. The European Nucleotide Archive accession number for the 1000 Genomes data is PRJNA262923. The Population Architecture using Genomics and Epidemiology (PAGE) dataset can be found on dbGap with accession number phs000356.v2.p1. Genotyping data for the KhoeSan population can be accessed on application to the Stellenbosch University Health Research Ethics Committee with reference to study N11/07/210.

## Abbreviations

GAI: Global ancestry inference
LAI: Local ancestry inference
SAC: South African coloured
SNP: Single nucleotide polymorphism
LAMP: Local ancestry in admixed populations
LD: Linkage disequilibrium
kb: Kilobase
EM: Expectation maximization
RMSE: Root mean square error
