# Supplementary material for: Putting RFMix and ADMIXTURE to the test in a complex admixed population
Source: BMC Genet. 2020 Apr 7;21:40. doi: 10.1186/s12863-020-00845-3 (PMC7140372; doi:10.1186/s12863-020-00845-3)
Supplement: Supplementary file 1 — Additional file 1: Table S1. Demographic model used to simulated SAC population [file 12863_2020_845_MOESM1_ESM.docx]

| **Generation** | **Reference Population** | | | | | |
| --- | --- | --- | --- | --- | --- | --- |
|  | Admixed | Bantu-Speaking African | European | South East Asian | East Asian | KhoeSan |
| 1 | 0 | 0.032 | 0 | 0 | 0 | 0.968 |
| 2 | 0.922 | 0.033 | 0.024 | 0.014 | 0.007 | 0 |
| 3 | 0.922 | 0.033 | 0.024 | 0.014 | 0.007 | 0 |
| 4 | 0.922 | 0.033 | 0.024 | 0.014 | 0.007 | 0 |
| 5 | 0.922 | 0.033 | 0.024 | 0.014 | 0.007 | 0 |
| 6 | 0.922 | 0.033 | 0.024 | 0.014 | 0.007 | 0 |
| 7 | 0.922 | 0.033 | 0.024 | 0.014 | 0.007 | 0 |
| 8 | 0.922 | 0.033 | 0.024 | 0.014 | 0.007 | 0 |
| 9 | 0.922 | 0.033 | 0.024 | 0.014 | 0.007 | 0 |
| 10 | 0.922 | 0.033 | 0.024 | 0.014 | 0.007 | 0 |
| 11 | 0.922 | 0.033 | 0.024 | 0.014 | 0.007 | 0 |
| 12 | 0.922 | 0.033 | 0.024 | 0.014 | 0.007 | 0 |
| 13 | 0.922 | 0.033 | 0.024 | 0.014 | 0.007 | 0 |
| 14 | 0.922 | 0.033 | 0.024 | 0.014 | 0.007 | 0 |
| 15 | 0.922 | 0.033 | 0.024 | 0.014 | 0.007 | 0 |

**Table S1: Demographic model used to simulated SAC population**
